# Supplementary material for: The impact of premature extrauterine exposure on infants’ stimulus-evoked brain activity across multiple sensory systems
Source: Neuroimage Clin. 2021 Dec 10;33:102914. doi: 10.1016/j.nicl.2021.102914 (PMC8683775; doi:10.1016/j.nicl.2021.102914)
Supplement: Supplementary data 1 [file mmc1.docx]

**Supplementary Information**

**The impact of premature extrauterine exposure on infants’ stimulus-evoked brain activity across multiple sensory systems**

**Schmidt Mellado et al.**

**Supplementary figures**


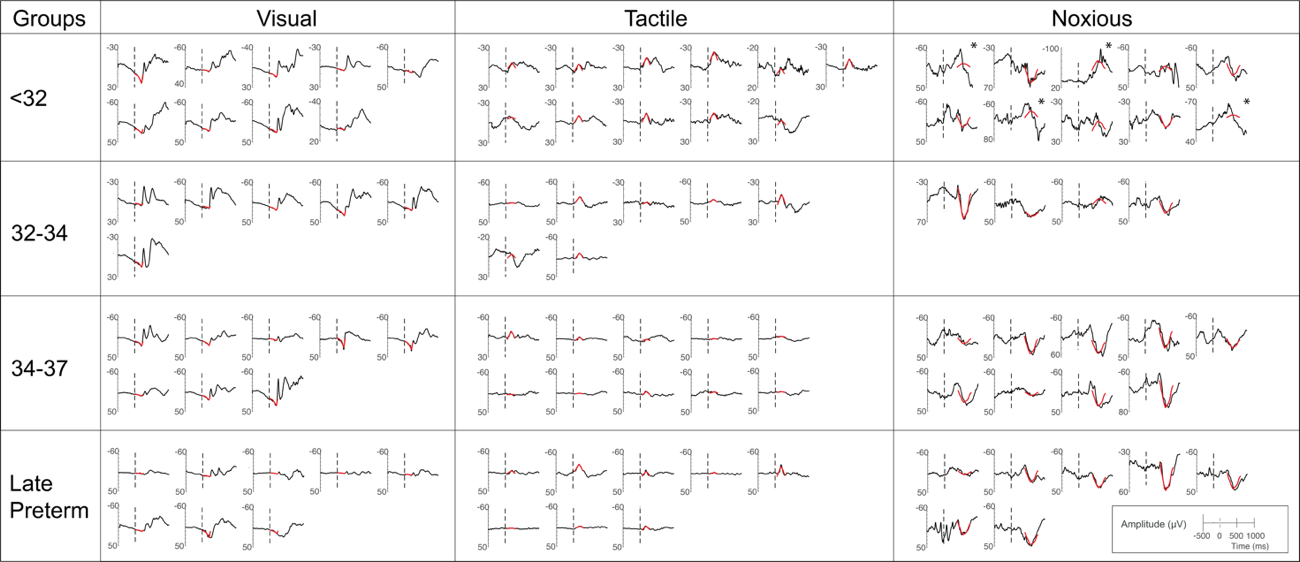


***Supplementary Figure 1: Individual EEG evoked responses for the different modalities separated by infant cohort.*** *Timeseries shows the subject****-****averaged tactile- and visual-evoked EEG responses and single trial noxious-evoked EEG responses for each recording included in the analysis. Data are displayed 500 ms pre-stimulus and 1000 ms post-stimulus. Vertical dotted lines denote the point of stimulus application, and the template for each modality is overlaid in red. * denotes the presence of delta brush.*
